# Immediate and delayed micro-tensile bond strength of different luting resin cements to different regional dentin

**DOI:** 10.7555/JBR.27.20120028

**Published:** 2012-12-06

**Authors:** Abdelraheem Mohamed Ali, Ibrahim Mohamed Hamouda, Mohamed Hamed Ghazy, Manal Mohamed Abo-Madina

**Affiliations:** a Department of Conservative Dentistry, Faculty of Dentistry, Mansoura University, Mansoura, Dakahleya 35516, Egypt;; b Department of Dental Biomaterials, Faculty of Dentistry, Mansoura University, Mansoura, Dakahleya 35516, Egypt;; c Department of Conservative Dentistry, Faculty of Dentistry, Umm Al Qura University, Makkah 715, Saudi Arabia;; d Crown & Bridge, Department of Conservative Dentistry, Faculty of Dentistry, Mansoura University, Mansoura, Dakahleya 35516, Egypt.

**Keywords:** immediate and delayed micro-tensile bond strength, luting resin cements, regional dentin

## Abstract

We sought to evaluate immediate and delayed micro-tensile bond strength of Panavia F2.0 and Multilink Sprint resin cement to superficial, deep and cervical dentin. Thirty-six freshly extracted non-carious human molars were sectioned in the mesiodistal direction to expose three different dentin regions including superficial dentin (1 mm below the dentine-enamel junction), deep dentin (1 mm above the highest pulp horn) and cervical dentin (0.5 mm above the cemento-enamel junction and 0.5 mm below the dentine-enamel junction). Resin cements were applied on dentin surfaces and composite blocks were luted under constant seating pressure. Each group was divided into three subgroups according to time intervals. Specimens were sectioned to obtain sticks of 1 mm^2^ in diameter and subjected to microtensile bond strength testing at a cross head speed of 1 mm/min. Both resin cements showed higher micro-tensile bond strength to superficial dentin than that to deep or cervical dentin (*P* < 0.001). Micro-tensile bond strengths of Panavia F2.0 were higher than those of Multilink Sprint at different dentin regions (*P* < 0.001). Immediate micro-tensile bond strengths were higher than those of delayed micro-tensile bond strengths for both resin cements (*P* < 0.001). It was concluded that resin cements with different chemical formulations and applications yield significantly different micro-tensile bond strengths to different dentin regions.

## INTRODUCTION

The success of adhesion procedures depends on adequate infiltration of monomers into demineralized collagen network, providing a hybrid layer formation, preventing restoration dislodgement and filling tooth structure[1]. However, structural complexities of dentin such as variation in permeability and adhesion of tubule orientation on substrate are still limiting factors for long-term stability of adhesive restoration[2].

Durability of dentin bonding is one of the most important issues of recent adhesive materials. The bond strength of different solvent-based adhesive systems gradually decreases over time, regardless of variable moisture pattern used for bonding procedure. Several studies have already reported significant reductions in bond strength values when stick-like specimens were immersed in water for periods similar to or higher than 6 months[3]–[5]. The immediate bonding effectiveness of the most current adhesive systems is quite favorable, regardless of the adhesive used. However, when these adhesives are tested in a clinical trial, the bonding effectiveness of some materials appears dramatically low, whereas bonds of other materials are more stable[6]. Long-term studies are considered to be the ideal method to validate the efficiency of restorative and adhesive materials. Therefore, it is desirable to develop an accelerated aging mode for challenging the durability of resin-dentin bonds in a relatively short period of time. One approach to this is to divide bonding specimens into smaller portions to decrease diffusion distances as suggested by Shono et al.[7]. Thus, immediate and long-term bond strength evaluations are necessary for product evaluation.

Dual-curing resin cements are polymerized by light and chemical polymerization. These two polymerization mechanisms form the basis for widespread use of these luting materials for definitive cementation of all-ceramic as well as composite and metal-based indirect restorations. Furthermore, dual polymerizing resin cements are characterized by high mechanical strength and excellent esthetic properties[8]. However, resin cement requires skillful handling when removing excess cement, especially during the time-consuming bonding procedure. The use of resin cement in clinical practice is complicated and technique sensitive[9],[10].

Recently, self-adhesive resin cements without surface pre-treatment has been introduced. These self-adhesive universal resin cements contain an acidic adhesive monomer which is stably integrated into composite matrix and is responsible for self-adhesive properties[10]. Currently, the choice of a luting material is based on the type of restoration and preparation. However, it is important to better understand the interaction between different dentin locations and type of luting materials[11].

More and more studies[7],[11],[12] have used micro−tensile measurement to evaluate dentin bond strength of adhesive system. The number of defects in a specimen made of a homogenous brittle material affects the tensile−strength characteristic. The stress is concentrated at the defected areas when the specimens are loaded, which initiate crack formation. The small adhesive interface used in the micro−tensile test contains fewer defects compared with larger interfaces, resulting in higher recorded bond strengths compared with other test methods that use larger surface area[11]. Also, this technique can be used to detect regional difference in resin−dentin bond strengths due to its use of small bonding areas[11],[12]. Nevertheless, as significant differences existed among different luting materials, the choice of a luting material should be based on the type of preparation and restoration as well as the need for fluoride release[11].

Resin luting agents should provide bond strengths sufficient to resist stress generated by its polymerization shrinkage. However, adhesive ability can be influenced by the variation in dental substrates where the adhesive materials are bonded[13]. The null hypothesis of the present study was that bond strength between resin luting agents and dental structures could be decreased when stored for 6 months. Additionally, the bond strength depends on the location of application. Therefore, we sought to evaluate the micro-tensile bond strength of two luting resin cements to different regional dentin surfaces after storage for 24 hours and 6 months either in stick form or block specimens before slicing into sticks.

## MATERIALS AND METHODS

### Reagents

The materials used in this study are presented in ***Table 1***. Thirty-six freshly extracted intact lower molars were selected. The study protocol was approved by the local institutional review board at Mansoura University. The experimental procedures were carried out strictly in accordance with the ethical committee of the Faculty of Dentistry of the authors' affiliated institutions. The teeth were free of any breakage as determined at fewer than 30×magnification by binocular stereo microscope (LOMO SF-100, MBC, Russia). Attached soft tissue and calculi were removed from the teeth by hand scaler. The teeth were stored in 0.5% chloramines-T solution for 2 weeks, and then prepared in distilled water at 4°C. The teeth were used within 3 months after extraction. In this study, the age difference among the collected teeth was ignored since a previous study showed that age did not greatly influence dentin bond strength[13].

**Table 1:** Materials used in this study

Materials and batch number	Composition	Manufacturers
Multilink Sprint #:598181AN	Dual-curing self-adhesive resin cement Dimethacrylaes; adhesive monomer; filler; initiators; stabilizers	Ivoclar Vivadent, Liechtenstein, Germany
Panavia F2.0 # 482KA	Dual-cured resin based cement	Kurary Medical Inc; Tokyo, Japan
ED Primer 2.0	Primer A: HEMA, MDP, 5-NMSA, water, acceleratorPrimer B: 5-NMSA, accelerator, water, sodium benzene sulphinate	
Base paste	Dual curing two step self etching luting resin hydrophobic aromatic and aliphatic dimethacrylate, sodium aromatic sulphinate,N,N-diethanol-p-toluidine, functionalized sodium fluoride, silanized barium glass	
Catalyst paste	MDP, hydrophobic aromatic and aliphatic dimethacrylate, hydrophilic dimethacrylate, silanized silica, photoinitiator, dibenzoyl peroxide	

The teeth were sectioned in the mesiodistal direction with a slow-speed water-cooled diamond disc to expose 72 flat buccal and lingual halves. Three different dentin regions including the superficial, deep and cervical regions were exposed. Superficial dentin was 1 mm below the dentino-enamel-junction. Deep dentin was 1 mm above the pulp horn. Cervical dentin was located 0.5 mm above the cemento-enamel-junction and 0.5 mm below the dentino-enamel-junction. Six hundred grit silicon carbide papers were used to produce standard smear layer of dentin. Each tooth specimen was embedded in acrylic resin (Acrostone, Cairo, Egypt) from its buccal or lingual side leaving the exposed dentin upward using special plastic mould. A light-activated resin composite material (Tetric-ceram, Ivoclar-vivadent Liechtenstein, Germany) was condensed in specially designed Teflon mould in 2 mm layers to produce composite block of 3 mm in width, 9 mm in length and 6 mm in height. Each layer was light-activated for 40 s with light curing unit (Litex 680A, Dentamerica Inc., City of Industry, CA, USA). The light curing unit was in the range of 350-520 nm in wavelength.

The intaglio surface of each composite block was ground with 180-grit SiC paper, cleaned with ethanol and dried with oil free air. The resin cement was applied on different regions of dentin (superficial, deep and cervical) according to the manufacturer's direction. Equal amounts of ED primer 2.0 A and B from Panavia F2.0 were mixed and applied to the dentin surface with a brush, and then left undisturbed for 30 seconds and dried with a gentle air flow. Equal amounts of base and catalyst were mixed for 20 seconds and then applied onto the primed substrate. For Multilink Sprint, with no conditioning steps, it was extruded from automix syringe and the desired quantity was applied directly onto the dentin.

By using a special loading device, the composite block was placed under a constant seating pressure of 3.0 kg that was maintained for 1 minute. When Panavia F2.0 was used, Oxyguard II was applied around resin cement to ensure complete anaerobic polymerization. Light-curing was then performed from four directions for 20 second along the cement interface using light curing unit. The teeth halves were divided into two main groups of 36 specimens according to resin cement used. Each group was divided into three subgroups of 12 specimens according to method of storage. For subgroup A, specimens were stored in distilled water at 37°C for 24 hours, and then sectioned to obtain sticks of 1 mm×1 mm×6 mm to be tested immediately. For subgroup B, specimens were stored in water for 6 months, and then sectioned into sticks and tested. For subgroup C, specimens were stored in distilled water at 37°C for 24 hours, and then sectioned into sticks that were stored in water for 6 months before testing.

**Fig. 1:**
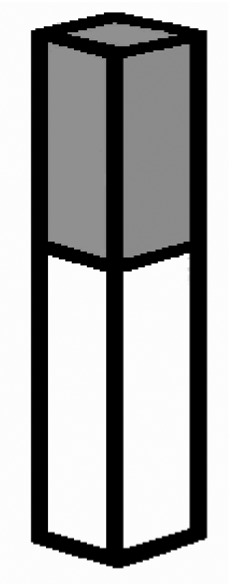
Schematic diagram of the tooth-composite stick.

The specimens were sectioned perpendicular to the adhesive-tooth interface using a low-speed diamond disc (0.5 thickness) under water cooling to produce dentin-resin composite sticks of 1×1 mm^2^ cross sectional area (***Fig. 1***). The dimensions of exact sticks were measured using a caliper (Iwanson, Martin, Germany) before being fixated into the gripping device. Each subgroup was divided into three divisions including sticks from superficial dentin, sticks from deep dentin, and sticks from cervical dentin.

Each dentin-composite stick was attached to a universal testing machine (Lloyd LRX, Type 500, Lloyd Instruments Ltd, Fareham, UK) using specially designed grip. The grip was made of two stainless steel articulating parts which were attached to each other at one end by a 0.35 mm thick brass sheet. This attachment permitted hinge movement of the two parts and ensured application of a pure microtensile force to the sticks. The sticks were glued to the free ends of the device using cyanoacrylate adhesive and subjected to tensile force at a cross head speed of 1 mm/min. The force was applied to the lower member via a steel ball, which loosely fitted in an outlet in the upper member. The pitch distance from the ball to the hinge was 80% of the distance from the specimen to hinge and in order to obtain the force exerted on the specimen. The measured force had to be multiplied by a value of 0.80. The values were corrected to the small force of 0.3 to 0.5 N composed of the weight of the articulating member and the spring action of the brass sheet[14]. The recorded force (Newton) was divided by the surface area of the specimen (mm^2^) to obtain micro-tensile bond strength (MPa).

### Scanning electron microscopy

Representative specimens were prepared for evaluation of the mode of failure under scanning electron microscope (SEM, JXA-840A Electron Probe Microanalyzer, Joel, Japan). The debonded dentin specimens were air-dried for 24 hours, gold-sputtered in an argon sputter coater (S150A sputter coater, Edward, England) for 2 minutes and observed by SEM to evaluate the failure modes.

### Statistical analysis

The statistical analysis of the data was performed using two-way ANOVA (with confidence interval of 95%) to determine the significance among the tested groups. Tukey's post-hoc test was used for multiple comparisons between the groups. *P* values <0.05 were considered statistically significant.

## RESULTS

The results of microtensile bond strength of tested materials to human dentin at different regions are presented in ***Table 2***. One-way ANOVA showed a significant difference among the tested groups (*P* < 0.001). The statistical analysis of the results showed a significant difference in microtensile bond strengths of Panavia F2.0 and/or Multilink Sprint after 24 hours and the other tested groups at the superficial dentin regions (*P* < 0.001). At deep and cervical dentin regions, Panavia F2.0 and Multilink Sprint showed significantly higher microtensile bond strengths than those of the other tested groups (*P* < 0.001). Both resin cements showed no significant differences between the groups stored for 6 months in sticks or as blocks before slicing at all dentin regions (*P* > 0.05).

**Table 2:** Microtensile bond strength (µTBS) of test groups to human regional dentin

Superficial	24-hours storage		Blocks stored for 6 months		Sticks stored for 6 months	
	Panavia F2.0	Multilink Sprint	Panavia F2.0	Multilink Sprint	Panavia F2.0	Multilink Sprint
Dentin	26.7^A,a^±4.2	10.1^C,a^±1.9	16.5^B,a^±2.8	4.5^D,a^±0.7	14.8^B,a^±2.6	3.9^D,a^±0.7
Deep dentin	10.7^A,b^±3.5	07.4^B,b^±1.2	07.5^B,b^±2.1	3.4^C,b^±0.6	07.6^B,b^±1.4	2.7^C,b^±0.7
Cervical dentin	09.3^A,b^±1.8	07.1^B,b^±1.0	06.5^B,b^±1.1	3.2^C,b^±0.4	05.7^B,b^±1.0	2.9^C,b^±0.5

Means in each row with different superscripted capital letters are significantly different at *P* < 0.05.

Means in each column with different superscripted small letters are significantly different at *P* < 0.05.

Panavia F2.0 showed no significant difference at dentin regions either after 24 hours, stored as blocks before slicing or stored as sticks for 6 months (*P* > 0.05). Multilink Sprint showed significantly higher microtensile bond strengths at the superficial dentin region than those at the other dentin regions either stored for 24 hours, as sticks or as blocks before slicing for 6-months (*P* < 0.05). Multilink Sprint showed no significant differences in microtensile bond strengths at deep or cervical dentin regions either stored for 24 hours, as sticks or as blocks before slicing for 6 months (*P* > 0.05).

### Scanning electron microscopy

**Fig. 2:**
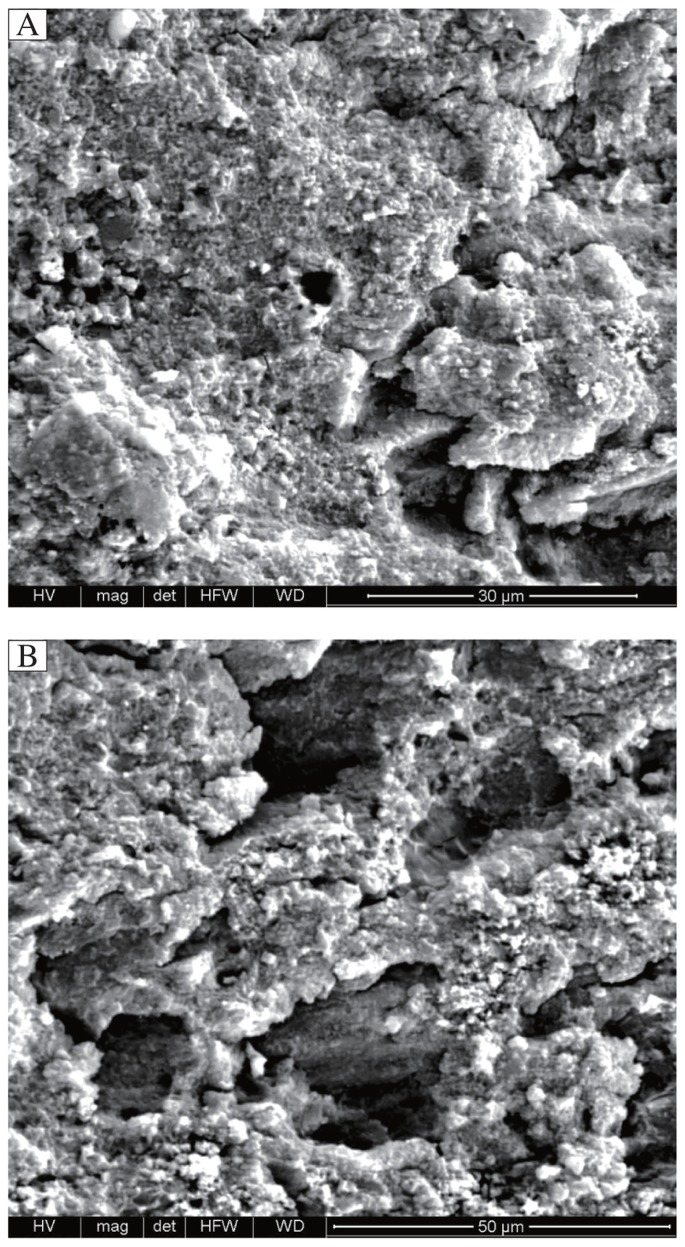
Scanning electronic microscopy showing the failure pattern exhibited after microtensile bond strength of fractured beams treated with Panavia F2.0 (4,000×). A: specimens stored for 24 hours. B: specimens stored for 6 months. The fractured surface showed adhesive/cohesive failure at the top of the hybrid layer after 24 hours &6 months at 26.7 Mpa & 16.5 Mpa respectively. The consistent layer of adhesive resin still sealing the dentinal tubules. Higher magnification of A&B, showed resin tags, which appear with porous structure and cracks.

The scanning electron microscopic observations of the fractured dentin surfaces of Panavia F2.0 specimens are showed in ***Fig 2***. Specimens of superficial, deep and cervical dentin surfaces treated with Panavia F2.0 and stored for 24 hours showed adhesive/cohesive failure at the top of the hybrid layer with cohesively fractured resin tags occluding the tubules which failed at 26.7 Mpa. Additionally, the consistent layer of adhesive resin still sealed the dentin side of the sample. At the higher dentin bond strength, a higher frequency of mixed type of failure was observed. There was a very tight relationship between adhesive and dentin. The failures were mainly mixed, from cohesive in dentin to cohesive in resin cement after 24 hours of storage (***Fig. 2A***). After 6-months of storage, Panavia F2.0 showed adhesive/cohesive failure of the hybrid layer with predominantly cohesive failure at 16.5 Mpa. The higher magnification 4,000×demonstrated interfacial failure that was typically mixed (areas of failed adhesive resin, cohesively failed adhesive and areas of cohesively failed dentin). The adhesive failure showed opening of dentinal tubules (***Fig. 2B***).

**Fig. 3:**
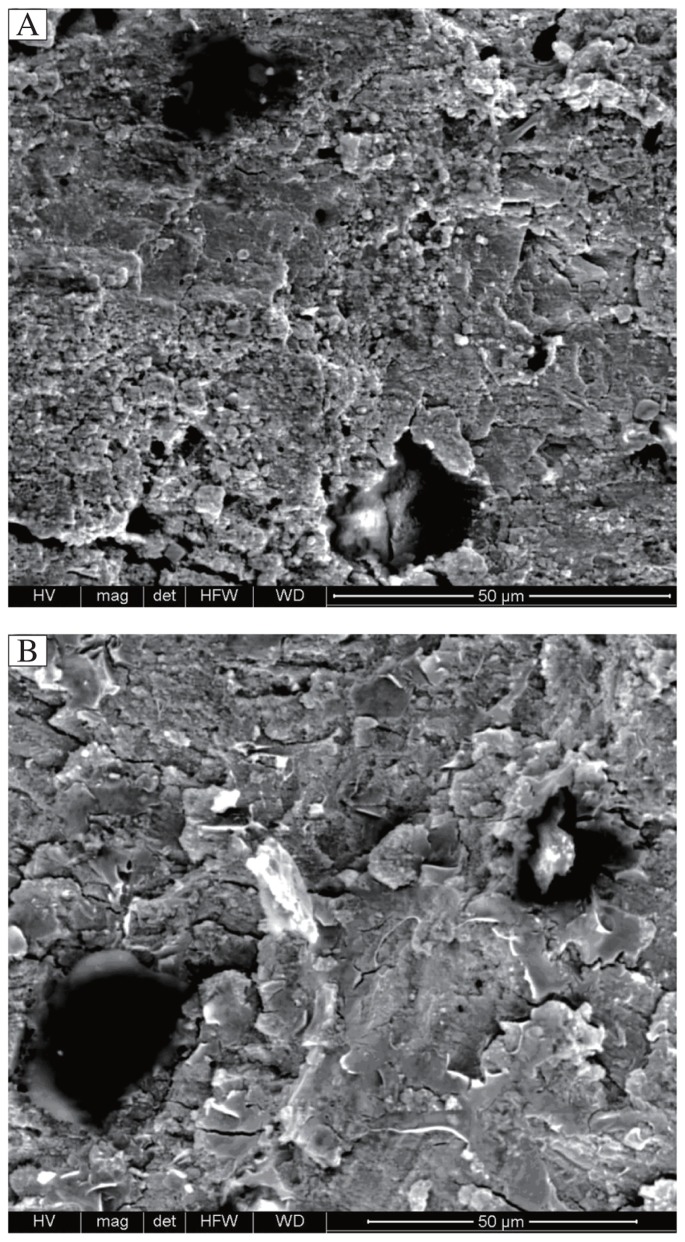
Typical SEM micrograph of fractured beams treated with Multilink Sprint (4,000×). A: specimens stored for 24 hours. B: specimens stored for 6 months. The fractured surface showed adhesive/cohesive failure at the top of the demineralized deep dentin after 24 hours &6 months at 10 Mpa & 4.5 Mpa respectively. The consistent layer of adhesive resin still sealing the dentinal tubules. Higher magnification of A&B, showed resin tags, which appear with porous structure and cracks.

Scanning electron microscopic analysis of the beams treated with Multilink Sprint are presented in ***Fig 3***. Specimens of superficial, deep and cervical dentin surfaces treated with Multilink Sprint and stored for 24 hours showed adhesive/cohesive failure at the top of the demineralized deep dentin surface with cohesively fractured resin tags occluding the tubules which failed at 10 Mpa. Additionally, the consistent layer of adhesive resin still sealed, the dentinal tubules of the samples. The fractured beams showed predominant cohesive failure after 24 hours of storage (***Fig. 3A***). After 6 months, samples treated with Multilink Sprint adhesive showed adhesive/cohesive failure with predominantly cohesive failure at 4.5 Mpa. An uneven adhesive layer was detected and empty tubules are shown at higher magnification 4,000×. Hybrid layer with some resin tags and empty tubules are shown (***Fig. 3B***). The fracture pattern distributions after the bond strength test indicated that bond failure during the early storage period occurred more frequently at the dentin-adhesive interface. With prolonged storage, the incidence of cohesive failure of dentin and/orresin increased.

## DISCUSSION

To some extent, the null hypothesis of the present study was accepted. The micro-mechanical bond is the main mechanism for bonding resins to dental substrates. This bond to dentin would occur through the infiltration of resin monomers in acid-etched dentin surfaces, presenting the total etch bonding concept, as a natural evolution of this technique[15],[16]. The ability to demineralize and infiltrate simultaneously the dentine surface, which utilized a phosphoric acid ester incorporated to hydrophilic and hydrophobic monomers, is the basis for the concept of self-etching systems. These systems simplify the bonding process and reduce the risk of incomplete infiltration through the collagen network exposed by demineralization[17].

In the present study, microtensile bond strength of self-etching resin cement (Panavia F2.0) to different dentin regions was higher than that of the self-adhesive resin cement (Multilink Sprint). The decreased micro-tensile bond strength of the self-adhesive resin cement may be attributed to the limited demineralizing action, yielding a superficial and irregular interaction with hard dental tissue. Relatively high viscosity and the moderate etching potential do not favor a deep infiltration of the resin into the collagen network, to generate an evident hybrid layer[7],[18].

In this study, ED primer 2.0 did not completely remove smear plugs. Therefore, Panavia F2.0 luting resin probably penetrated into residual smear plug to partially demineralized collagen network around tubular walls to form a thin bonding interface. However, this thin bonding of Panavia-F2.0 to the walls of tubules was strong enough to make hybridized smear plugs and resin tags fracture at the tubule orifice during µTBS testing instead of being pulled out from the tubules. It could be concluded that the top of hybridized smear layer became the weak link during µTBS testing. Compared to Panavia-F2.0, a rather high percentage of partial adhesive failure that left a thin layer of cohesively fractured luting resin was found in all Multilink Sprint groups, indicating that the adhesion of luting resin to dentin was weak.

In the present study, µTBS of both resin cements to superficial dentin were significantly higher than those to deep dentin and cervical dentin. This was attributed to superficial dentin that there was more inter-tubular dentin area rich in collagen fibrils than in deep and cervical dentin. Therefore, µTBS was significantly higher in superficial dentin due to the opportunity of more micromechanical adhesion to collagen fibrils in the hybrid layer.

Theoretically, in deep and cervical dentin the decreased amount of inter tubular dentin available limits the contribution of the hybrid layer to the µTBS, while the increased number and diameter of the tubules increases the cross-sectional area and volume of the resin tags. Therefore, the cohesive strength of the resin tags and its hybridization to tubular walls play an important role in determining bond strength in deep dentin[4]. In cervical dentin, resin tags penetrated into oblique tubules to provide non-parallel retention. This might account for a differing µTBS in cervical dentin compared to deep dentin[19].

In order to obtain reliable initial tensile bond strengths of the two luting resins to dentin, the storage condition was at least 24 hours. The curing degree of resins is an important factor influencing bond strength. For Panavia F2.0, the radical polymerization reaction should be almost completed and stable after 24 hours water storage[7]. Early bond strength is considerably important, since the bond must be capable of withstanding the high tensions arising from polymerization shrinkage of the restorative composites. Otherwise, these tensions would break up the interface, leading to gap formations and, consequently, to postoperative sensitivity and secondary caries[13]. Storage in water may result in hydrolytic degeneration of the interface components, and especially of the resin and /or collagen[20]. Hydrolysis is a chemical process that breaks covalent bonds between the polymers by addition of water to ester bonds, resulting in loss of the resin mass. This is considered as one of the main reasons for resin degradation within the hybrid layer, contributing to the reduction in bond strengths created by dentin adhesives over time[21].

The results of this study have shown significant reduction in bond strength values when stick-like specimens or restored tooth halves were maintained immersed in water for 6 months before slicing. This may be attributed to the surface/volume ratio which was significantly lower for the whole restoration than for the individual sticks obtained from a similar size restoration. Water diffusion occurs slowly from the periphery to the inner region, making the outer surface more susceptible to water degradation. It may be the reason why the specimens stored for 6 months as sticks showed larger decrease in bond strength indexes than that stored as blocks before slicing into stick specimens, which remained more stable[22].

In conclusion, luting resins with different chemical formulations and applications yield significantly different bond strengths to different regions in human dentin. Bonding to superficial dentin was higher than that to deep and cervical dentin. Water plays an important role in resin-dentin bond degradation.
